# Evaluating the relationship between right-to-left shunt and white matter hyperintensities in migraine patients: A systematic review and meta-analysis

**DOI:** 10.3389/fneur.2022.972336

**Published:** 2022-08-18

**Authors:** Joshua Y. P. Yeo, Claire X. Y. Goh, Ying Kiat Tan, Bryan T. S. Sim, Beverly L. X. Chan, Nicholas L. Syn, Yinghao Lim, Amanda C. Y. Chan, Vijay K. Sharma, Jonathan J. Y. Ong, Leonard L. L. Yeo, Ching-Hui Sia, Benjamin Y. Q. Tan

**Affiliations:** ^1^Department of Medicine, National University Health System, Singapore, Singapore; ^2^Yong Loo Lin School of Medicine, National University of Singapore, Singapore, Singapore; ^3^Department of Cardiology, National University Heart Centre, Singapore, Singapore; ^4^Division of Neurology, Department of Medicine, National University Health System, Singapore, Singapore

**Keywords:** migraine, migraine with aura, white matter, patent foramen ovale, ultrasonography, doppler, transcranial, arteriovenous malformations

## Abstract

**Introduction:**

White matter hyperintensities (WMHs) have been observed with greater frequency in patients with migraine and are thought to be associated with impaired cognition and function. The relationship between WMHs and right-to-left shunt (RLS) in migraine patients is unknown. We performed a systematic review to determine if there is an association between RLS and WMHs in patients with migraine.

**Methods:**

A systematic search of the literature was performed in PubMed and Embase using a suitable keyword search strategy from inception up to 16th June 2021. All studies that included patients with migraine and studied RLS and WMHs were included.

**Results:**

A total of 8 non-randomized observational studies comprising 1125 patients with migraine were included; 576 had an RLS, compared to 549 patients with no RLS. The mean age of the study populations ranged from 28.4 to 43 years, while the average duration from migraine diagnosis ranged from 5.1 to 19 years. The proportion of female to male patients was consistently higher in all studies (60.0–94.4%). Amongst migraine patients with RLS, 338 patients (58.7%) had WMHs. In contrast, 256 (46.6%) of migraine patients without RLS had WMHs. RLS was significantly associated with the presence of WMHs in migraine patients (OR: 1.56, 95% CI: 1.05–2.34, *p* = 0.03).

**Conclusion:**

In migraine patients, RLS was significantly associated with the presence of WMHs. Longitudinal studies are warranted to establish RLS as a risk factor for WMHs in patients with migraine, and to establish the significance of these changes.

## Introduction

White matter hyperintensities (WMHs) have been observed with greater frequency in patients with migraine. A recent review showed that in a patient population of <50 years old without risk factors, WMH prevalence of up to 70% was noted in patients with migraine with a 3.9-fold increase in the odds of WMH being present compared with controls. This effect was less obvious in population-based studies that included patients up to 74 years old or only included patients older than 55 years old, leading the reviewers to conclude that the effect of migraine on developing WMH may be overwhelmed by other risk factors in middle age (1). An earlier study that used a 1.5T magnetic resonance (MR) scanner reported a 39% prevalence (2); later studies with higher resolution MR imaging with 3T showed a prevalence closer to 70% in patients with migraine without traditional cardiovascular risk factors (3, 4). Two longitudinal studies also showed more rapid progression of deep WMHs in female patients with migraine compared to controls (5, 6). WMHs are inversely associated with mobility, cognition and function (7). In a recent study, baseline WMHs were found to be associated with poorer functional status and cognition as measured by mini-mental state examination (MMSE), while progression of WMHs was associated with a decrease in executive function score (8). Other types of migraines have also been evaluated for relationships with WMH; of interest, 1 population study was found which sporadic hemiplegic migraines (SMH) to patients with migraine and found no significant difference in the overall rate of WMH (9). This study did not review any association with vascular risk factors or RLS.

There is recent interest in the relationship between migraine, aura status and RLS (1, 10, 11). One hypothesis is that vasoactive substances bypass the pulmonary circulation to directly enter the systemic circulation in patients with RLS, inducing migraine attacks and the aura symptoms (12). While some studies reported an association of RLS with migraine with aura (2), other studies showed no difference in the prevalence of RLS between migraine subtypes (10, 11). Of the types of RLS, a patent foramen ovale (PFO) is the most common and is present in >25% of the healthy population (13), while other subtypes such as pulmonary arteriovenous malformations are rarer and associated with specific hereditary conditions (14). Accordingly, various clinical trials have sought to demonstrate a reduction in migraine frequency post-RLS closure. The MIST trial showed no significant difference between patients who had PFO closure vs. patients who had a sham procedure done (15). It is worth noting that the primary outcome studied was complete cessation of migraines and might not have been able to detect subtle improvements. Subsequent trials performed include the PRIMA and PREMIUM trials (16, 17); PRIMA failed to show a reduction in its primary endpoint (reduction in days with migraine) while PREMIUM showed a statistically significant reduction in headache days (secondary endpoint) but again failed to show a reduction in its primary outcome (responder rate with 50% reduction in migraine attacks).

Given the uncertain relationship between RLS and WMHs in patients with migraine, we performed a systematic review and meta-analysis of the literature to describe the prevalence of WMHs in migraine patients with or without RLS.

## Methods

### Search strategy

We conducted the systematic review in accordance with the Preferred Reporting Items of Systematic Reviews and Meta-Analysis (PRISMA) guidelines. A literature search was performed on PubMed and Embase for articles published from inception up to 16th June 2021. The search strategy consisted of combinations of the following search terms relating to migraines, (e.g. “migraine,” “migraine with aura,” “MWOA,” “migraine without aura”) white matter lesions (e.g. “white matter lesions,” “WML,” “WMH,” “white matter hyperintensit^*^,” “leukoenceplaopa^*^,” “leukoaraiosis,” “silent brain infarct^*^,” “SBI,” “ischemic brain lesions”) and RLS (e.g “patent foramen ovale,” “PFO,” “pulmonary arteriovenous malformations,” “RLS,” “RILES” and “right to left shunt”). The references from included studies were searched to detect studies missed by the electronic search. The search was performed by two independent reviewers (JY and CG), with any disagreements regarding study relevance resolved by a senior author (BT).

### Eligibility criteria

The study population included patients diagnosed with migraine. We included all studies that reported the prevalence of WMHs and evaluated the presence of RLS in patients with migraine. Identification and confirmation of WMHs using MR imaging was required. The presence of RLS was defined as any abnormal communication between right and left cardiac chambers including patent foramen ovale, atrial septal defects and pulmonary arteriovenous malformation. Seven of the studies employed contrast-enhanced transcranial Doppler (TCD) at rest and with Valsalva, while one study used echocardiographic data (precise modality not stated). All the studies except for one (not reported) employed a 1.5T MRI scanner for diagnosis of WMHs. Only studies published in the English language and included the full text (not conference proceedings) were included. All the studies that were included relied on the International Headache Society criteria for classification of migraines (18). We excluded studies that were reviews, case reports, case series, studies that studied pediatric populations, studies that did not diagnose WMHs on MRI, and studies that did not report RLS. The specific inclusion and exclusion criteria are detailed in Table 1.

**Table 1:** PECOS (Population Exposure Comparison Outcomes Study Design) table.

**PECOS**	**Inclusion criteria**	**Exclusion criteria**
Population	Patients with migraine	Stroke/pediatric population WMH not diagnosed on MRI No mention of RLS
Exposure	Presence of RLS (patent foramen ovale, pulmonary arteriovenous malformation, atrial septal defect)	
Comparison	Patients with migraine without any RLS	
Outcome	Prevalence of WMH	
Study design	Articles in English Published up to 16^th^ June 2021 Database: PubMed and Embase	Articles not available in English Conference abstracts or poster presentations for which full text unavailable Studies that were not observational cohort studies

WMH, white matter hyperintensities; MRI, magnetic resonance imaging; RLS, right-to-left shunt.

### Data extraction

Quantitative data collected was extracted from the included studies by two authors (JY and BS). Absolute numbers were included along with the percentages as appropriate. Where available, the data included study design, study population, duration of disease, type of migraine/WMHs and patient demographics (age and gender). We also included descriptions of how RLS or WMHs were diagnosed. Where data were reported in an incomplete fashion, we contacted the authors to obtain the relevant data.

### Risk of bias assessment

The quality and risk of bias of included studies were assessed using the GRADE Assessment Tool and the Newcastle Ottawa Scale respectively. The GRADE Assessment tool assesses quality of evidence in terms of study limitations, inconsistency, indirectness, imprecision and publication bias. The Newcastle Ottawa scale evaluates quality of evidence based on selection of study groups (4/5 points), comparability of groups (2 points), and ascertainment of exposure and outcomes (3 points). These were graded with the consensus of 3 researchers (YKT, CG & BT).

### Statistical analysis

We performed a random-effects meta-analysis on the odds ratio of outcome identified (prevalence of WMHs), comparing patients with migraine with and without RLS. Further analysis with pooled effect size estimates was performed on two studies which reported adjusted odds ratios. Numerical data points were presented with the absolute number or means as appropriate. Categorical variables were stated as percentages. Heterogeneity was assessed with the I^2^ statistic. All data analysis was conducted using the Cochrane Collaboration's Review Manager (RevMan 5.4) Software Package. A *p* < 0.05 was taken as the criterion for statistical significance.

## Results

### Study assessment and grading

**Figure 1:**
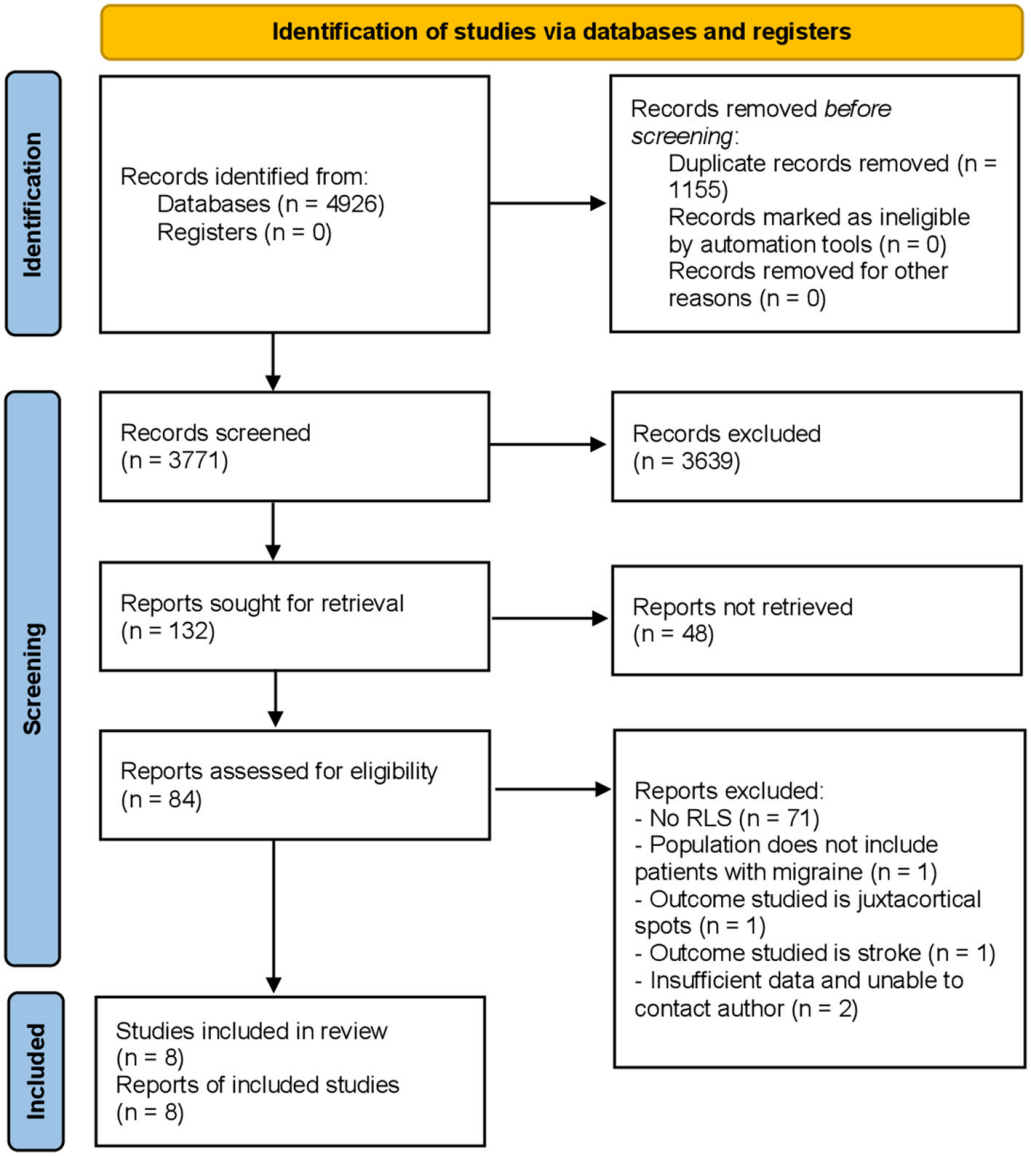
PRISMA 2020 flow diagram for new systematic reviews which included searches of databases and registers only.

A total of 4,926 studies were identified on initial search, of which 8 were finally selected (19–26) for analysis. The study selection process is illustrated in the PRISMA flowchart (Figure 1).

### Study characteristics

Among the 8 studies included for analysis, there were 5 cross-sectional studies, 2 case-control studies and 1 prospective cohort study. All studies included patients with migraine, but with different migraine subtypes. Three studies included only migraine with aura (22, 23, 25), one study included only migraine without aura (26), three studies included both migraine with and without aura (19, 20, 24), and one study did not specify the subtype of migraine (21).

A total of 1125 patients with migraine were included across all studies, with the number of patients in each study varying from 40 to 334. The mean age of the study populations ranged from 28.4 to 43 years, while the average duration from migraine onset ranged from 5.1 to 19 years. The proportion of female to male patients was consistently higher in all studies (60.0% to 94.4%). All studies diagnosed WMHs on MR imaging with at least 2 independent neurologists or neuroradiologists, except one study which did not specify. Characteristics of all the included studies are reported in Table 2. Of the 1125 patients, 576 (58.7%) of patients had a RLS. Three of the studies reported the specific type of RLS (Table 3) while the others left the type unspecified.

**Table 2:** Characteristics of included studies.

**References**	**Study design**	**Patients**	**Age (mean otherwise stated)**	**Females (%)**	**Duration of disease**	**Migraine type**	**WML diagnosed by**	**MRI magnet strength**	**Type of WMH reported**	**Analysis**
Del Sette et al. (23)	Cross-sectional study	80	37.2	Not reported	16.14	Migraine with aura	2 neuroradiologists	1.5T	Not specified	Mann–Whitney *U*-test Kruskal–Wallis ANOVA test Spearman rank order test
Rao et al. (24)	Case-control study	100	40	60 (60.0)	19	Both with and without aura	2 neuroradiologist	Not specified	Not specified	Pearson χ^2^ tests
Adami et al. (22)	Cross-sectional study	185	36	143 (77.3)	Not reported	Migraine with aura	1 neuroradiologist and 2 neurologists	1-1.5T	PV-WML, D-WML	Pearson χ^2^ Fisher exact, unpaired t, and Mann–Whitney *U*-tests Spearman rank correlation tests Logistic regression analysis
Park et al. (19)	Cross-sectional	242	28.4	183 (71.5)	5.1	Both with and without aura	2 neurologists	1.5T	D-WML	Pearson χ^2^ tests Unpaired Student's *T*-tests Bootstrapping methods Multiple binary regression tests
Dinia et al. (25)	Prospective study	41	41.8	33 (80.5)	16.9	Migraine with aura	2 neuroradiologists	1.5T	Not specified	Mann-Whitney *U*-test Fisher's exact test Pearson's correlation test
Uçar et al. (26)	Case-control study	40	36.2	37 (90.0)	WMH group: 8 Control group: 4	Migraine without aura	Not reported	1.5T	Not specified	Pearson χ^2^ test Fisher's exact tests Student t-test Mann–Whitney U-test Spearman correlation analysis
Iwasaki et al. (20)	Cross-sectional study	107	39.0 (median)	101 (94.4)	18.0 (median)	Both with and without aura	2 neurologists	1.5T	Not specified	Pearson χ^2^ tests Mann–Whitney U test Logistic regression
Jiang et al. (21)	Cross-sectional study	334	43.0	241 (72.2)	11.69	Not specified	2 neurologists	1.5T	D-WML and PV-WML	Pearson's χ^2^ test Unpaired *t*-tests Binary logistic regression models (odds ratio [OR], 95% confidence interval [CI]) for MRI outcomes

WMH, white matter hyperintensities; PV-WML, periventricular white matter lesions; D-WML, deep white matter lesions.

**Table 3:** RLS reported in included studies.

**References**	**Total patients**	**Presence of RLS**	**Number of RLS (percentage)**	**Method of diagnosis**	**Type of RLS reported**
Del Sette et al. (23)	80	36	45%	Contrast-enhanced transcranial Doppler at rest and with Valsalva, counting micro-bubbles in MCA	Not specified
Rao et al. (24)	100	41	41.0%	Transcranial doppler	Patent foramen ovale
Adami et al. (22)	185	114	61.6%	Contrast-enhanced transcranial Doppler at rest and with Valsalva, counting micro-bubbles in MCA	Not specified
Park et al. (19)	242	89	36.8%	Contrast-enhanced multifrequency -transcranial Doppler at rest and with Valsalva, counting micro-embolic signals (MES) with RLS defined as ≥ 11 MESs.	Not specified
Dinia et al. (25)	41	13	31.7%	Contrasted transcranial doppler	Not specified
Uçar et al. (26)	40	2	5%	Echocardiographic data	Atrial septal defect, inter-atrial septum
Iwasaki et al. (20)	107	57	53.2%	Contrast-enhanced transcranial Doppler at rest and with Valsalva, counting high-intensity transient signals	Patent foramen ovale, pulmonary arteriovenous malformations
Jiang et al. (21)	334	224	67%	Contrast-enhanced transcranial Doppler at rest and with Valsalva, counting micro-bubbles in MCA	Not specified

RLS, right-to-left shunts; MCA, middle cerebral artery.

### Prevalence of white matter hyperintensities

**Figure 2:**
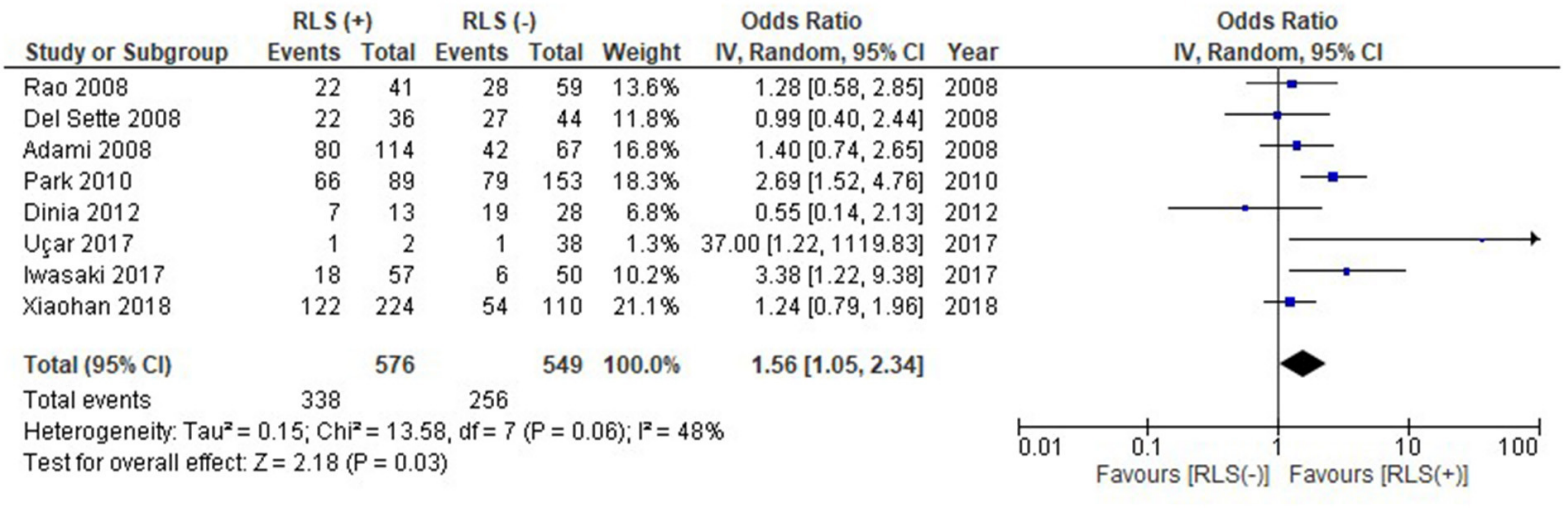
Forest plot of the association of RLS with prevalence of WMH (odds ratio).

**Figure 3:**
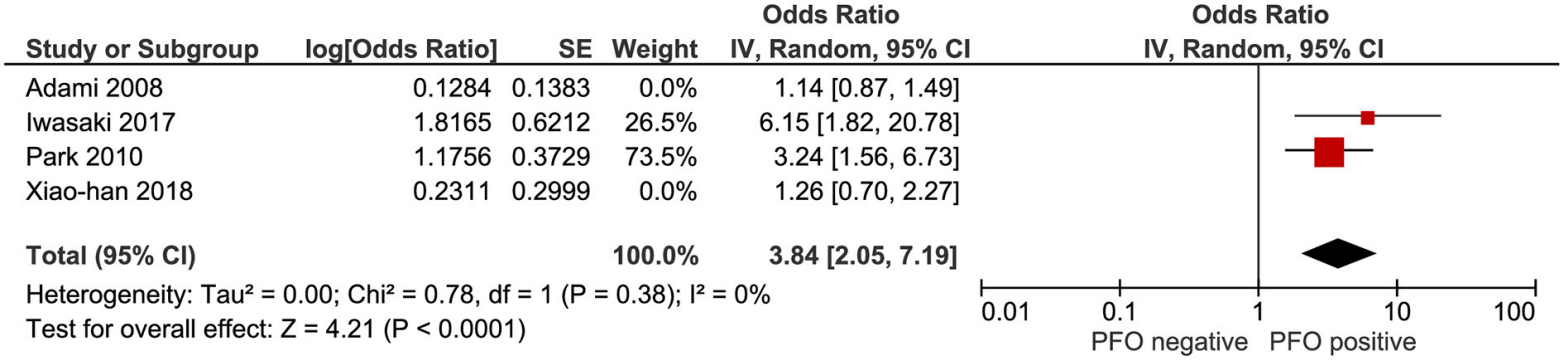
Forest plot of the association of RLS with prevalence of WMH (adjusted odds ratio).

With regards to prevalence of WMHs, a total of 1,125 patients across all 8 studies were assessed. Amongst migraine patients with RLS, 338 patients (58.7%) had WMHs. In comparison, 256 (46.6%) of migraine patients without RLS had WMHs. RLS was associated with a higher prevalence of WMHs in patients with migraine (OR: 1.56, 95% CI: 1.05–2.34), with low-moderate heterogeneity (I^2^ = 48%) as shown in Figure 2. To mitigate the effect of known confounders such as age, hypertension, smoking and sex, studies which reported adjusted odds ratios were analyzed for a pooled effect size estimate. As illustrated in Figure 3, the pooled effect size estimate for RLS showed that there was a significantly larger number of patients with WMHs in migraine patients with RLS, compared to those without RLS (OR: 3.84, 95% CI: 2.05–7.19).

### Quality assessment

Using the GRADE tool, the quality of evidence was assessed to be high for prevalence of WMHs (Supplementary Table 1). Following assessment with the Newcastle-Ottawa scale, the cross-sectional studies achieved 9–10 out of a maximum 10 points on the Newcastle-Ottawa scale, while the lone cohort study achieved 9 out of a maximum of 9 points on the Newcastle-Ottawa Scale. This signifies the high quality and low risk of bias for selection for these studies. However, the case control studies achieved 7–8 points out of a maximum 9 points on the Newcastle-Ottawa scale, suggesting a moderate risk of bias for selection (Supplementary Tables 2–4).

## Discussion

This study aimed to provide a comprehensive systematic review and meta-analysis of the available literature regarding prevalence of WMHs, and the mediating effect of RLS in patients with migraine. It demonstrates that there is a significant difference in the prevalence of WMHs in migraine patients with and without RLS.

RLS had previously been postulated as a possible mechanism of WMHs in patients with migraine (1). However, conflicting results have been reported. Five of the included studies suggested that RLS had no effect on the load or presence of WMHs in patients with migraine, while 3 studies did in fact show a statistically significant effect on the presence of WMHs (19–26). Our meta-analysis demonstrated a statistically significant difference with an odds ratio of 1.56 [95% CI, 1.05–2.34, *p* = 0.03], bolstering the case for RLS as a possible mechanism of WMHs in migraine patients. This effect was demonstrated as well with a pooled effect size estimate for the studies which reported an odds ratio adjusting for known confounders such as age, sex, smoking or hypertension.

Previous studies have noted an increase of up to 3.9 times in the odds of WMHs in patients with migraine compared to controls with a particularly strong effect in younger populations aged <50 with no vascular risk factors, possibly because vascular risk factors tend to overwhelm the relative contribution of migraine for the development of WMHs in older patients (10). Owing to the manner in which data was reported, we were unable to adjust for the effect of age in our study beyond the studies which reported an adjusted odds ratio.

Of interest, other studies have sought to show headache improvement in patients with migraine who undergo PFO closure (27, 28). It is postulated that incomplete transit of venous blood through the lung filter allows vasoactive substances to circumvent the filter and thus precipitate migraine attacks (29). Typically, headache improvement is assessed by clinical scoring systems such as the MIDAS questionnaire. Considering our study's findings, it may be worthwhile including outcomes related to WMH prevalence in PFO closure trials. A meta-analysis showed that there were significant associations between WMHs and incident stroke, incident dementia, global cognitive decline as well as mortality (30). WMHs may well be considered a suitable surrogate measure for these other clinical outcomes of stroke and cognitive decline. It may be appropriate to assess the effectiveness of PFO closure as a treatment in reducing incident stroke or cognitive impairment.

### Strengths and limitations

This study reveals a potentially interesting relationship between WMHs and RLS in the context of patients with migraine. WMHs have been associated with stroke, dementia and mortality (30). Our findings support an association with RLS and to our knowledge is the first systematic review to aggregate existing studies regarding RLS in patients with migraine and WMH burden. This effect held with a pooled effect size estimate. While we found a significant association between RLS and WMH in migraine patients, the effect size was moderate and causality cannot be interpreted due to the retrospective nature of the studies that were included in this systematic review. Further longitudinal studies are necessary to establish causality between RLS and WMH in this patient cohort.

One important limitation was that the type of RLS was not always reported in the included studies. Only 3 studies (20, 24, 26) specified the type of RLS. Another limitation was that not all studies reported if the WMHs were in the periventricular or deep regions, nor were they classified in a standardized manner. Only 2 studies presented the data for both deep and periventricular regions, whilst 1 study reported on deep WMHs, with the remaining studies leaving the type of WMHs unspecified. Previous studies have suggested that periventricular WMHs were associated with impaired cognitive function, but less so if they were in the deep locations (31). Future studies that differentiate between the location of WMHs found in association with presence of RLS may prove to be more illuminating with regards to the clinical implications of our findings.

A further limitation worth considering is that the quantitative data reported was not granular enough to be stratified according to variables such as age or gender. This was partially mitigated by applying an analysis of the pooled effect size estimate where published in the studies reviewed. It should also be noted that not all studies consistently reported on the number, volume, confluence, accrual over time and gadolinium enhancement of the white matter hyperintensity lesions. As such, differential diagnoses like demyelinating disorders including multiple sclerosis may have been neglected in this meta-analysis.

Finally, an important limitation lies in the way that RLS was diagnosed. All the studies detected RLS *via* TCD, except for Uçar 2017 as opposed to the gold standard of diagnosis which remains transesophageal echocardiography (TEE). Studies comparing TCD vs. TEE found a higher sensitivity for TEE (31–33); it is notable that most of the advantage for TEE came from minimal shunts which may not be clinically relevant. More recent studies have suggested that TCD may have comparable sensitivity of 94–100% (34, 35) and remains valuable as a complementary, non-invasive option for screening, while TEE remains the best modality for delineating anatomy and detection of other cardiac abnormalities such as atrial septal defects (36).

## Conclusion

In migraine patients, RLS was significantly associated with the presence of WMHs. Longitudinal studies are warranted to establish RLS as a risk factor for WMHs in patients with migraine, and to establish the significance of these changes.

## Data availability statement

The original contributions presented in the study are included in the article/Supplementary material, further inquiries can be directed to the corresponding author.

## Author contributions

JY: writing–original draft (lead), reviewing and editing (equal), investigation (equal), and formal analysis (equal). CG and YT: writing–original draft (supporting), reviewing and editing (equal), investigation (equal), and formal analysis (equal). BS: reviewing and editing (equal) and investigation (equal). BC, YL, AC, VS, and JO: reviewing and editing (equal). NS: reviewing and editing (equal) and methodology (equal). LY and C-HS: reviewing and editing (equal) and conceptualization (equal). BT: reviewing and editing (equal), methodology (equal), and conceptualization (equal). All authors contributed to the article and approved the submitted version.

## Funding

LY was supported by the National Medical Research Council (NMRC), Singapore (NMRC/MOH-TA19Nov-003). C-HS was supported by the National University of Singapore Yong Loo Lin School of Medicine's Junior Academic Fellowship Scheme.

## Conflict of interest

The authors declare that the research was conducted in the absence of any commercial or financial relationships that could be construed as a potential conflict of interest.

## Publisher's note

All claims expressed in this article are solely those of the authors and do not necessarily represent those of their affiliated organizations, or those of the publisher, the editors and the reviewers. Any product that may be evaluated in this article, or claim that may be made by its manufacturer, is not guaranteed or endorsed by the publisher.

## Abbreviations

WMH: white matter hyperintensities
RLS: right-to-left shunt
MR: magnetic resonance
PFO: patent foramen ovale
PRISMA: Preferred Reporting Items of Systematic Reviews and Meta-Analysis
TEE: transesophageal echocardiography.
