# Supplementary material for: Evaluating the relationship between right-to-left shunt and white matter hyperintensities in migraine patients: A systematic review and meta-analysis
Source: Front Neurol. 2022 Aug 18;13:972336. doi: 10.3389/fneur.2022.972336 (PMC9433673; doi:10.3389/fneur.2022.972336)
Supplement: Supplementary file 1 [file Table_1.docx]

**Supplementary Table: GRADE Assessment**

| **№ of studies** | **Certainty assessment** | | | | | | **Effect** | | | **Certainty** | **Importance** |
| --- | --- | --- | --- | --- | --- | --- | --- | --- | --- | --- | --- |
|  | **Study design** | **Risk of bias** | **Inconsistency** | **Indirectness** | **Imprecision** | **Publication bias** | **№ of events** | **№ of individuals** | **Rate (95% CI)** |  |  |
| Prevalence of WMH (assessed with Odds Ratio; Scale from: 0.01 to 100) | | | | | | | | | | | |
| 8 | Observational study. | not serious | serious^a^ | not serious | not serious | undetected | - | 1125 | 1.56  (1.05 to 2.34) | ⨁⨁⨁⨁ HIGH | CRITICAL |

#### Explanations

a. While there is significant heterogeneity as calculated by the I_2_ value (48%), it is worth noting that 3 of the 8 studies in the meta-analysis showed a trend that suggests increased incidence of WMH in patients with RLS, while another 3 of the 8 studies showed a significant increase in incidence of WMH in RLS patients. This suggests that the overall conclusion is unlikely to be affected by heterogeneity, as a total of 6 of the 8 studies showed the incidence of WMH to be higher in patients with RLS.
