# Supplementary material for: Evaluating the relationship between right-to-left shunt and white matter hyperintensities in migraine patients: A systematic review and meta-analysis
Source: Front Neurol. 2022 Aug 18;13:972336. doi: 10.3389/fneur.2022.972336 (PMC9433673; doi:10.3389/fneur.2022.972336)
Supplement: Supplementary file 2 [file Table_2.docx]

**Supplementary Table 2 (Cohort study)**

| **Author** | **Year** | **Selection**  **(Max ☆☆☆☆)** | | | | **Comparability**  **(Max ☆☆)** | **Outcome**  **(Max ☆☆☆)** | | |
| --- | --- | --- | --- | --- | --- | --- | --- | --- | --- |
|  |  | **Representativeness of the exposed cohort** | **Selection of the non-exposed cohort** | **Ascertainment of exposure** | **Outcome absent at study start** | **Comparable cohorts (design or analysis)** | **Assessment of outcome** | **Appropriate follow-up time** | **Adequate follow-up of cohorts** |
| Dinia, L | 2012 | ☆ | ☆ | ☆ | ☆ | ☆☆ | ☆ | ☆ | ☆ |
