# Supplementary material for: Evaluating the relationship between right-to-left shunt and white matter hyperintensities in migraine patients: A systematic review and meta-analysis
Source: Front Neurol. 2022 Aug 18;13:972336. doi: 10.3389/fneur.2022.972336 (PMC9433673; doi:10.3389/fneur.2022.972336)
Supplement: Supplementary file 3 [file Table_3.docx]

**Supplementary Table 3 (Case control studies)**

| **Author** | **Year** | **Selection**  **(Max ☆☆☆☆)** | | | | **Comparability**  **(Max ☆☆)** | **Exposure**  **(Max ☆☆☆)** | | |
| --- | --- | --- | --- | --- | --- | --- | --- | --- | --- |
|  |  | **Case definition** | **Representativeness of the cases** | **Selection of controls** | **Definition of controls** | **Comparable cases and controls (design or analysis)** | **Ascertainment of exposure** | **Same method of ascertainment for cases and controls** | **Non-response rate** |
| UÇAR, C.A. | 2017 | ☆ |  |  | ☆ | ☆☆ | ☆ | ☆ | ☆ |
| Rao, R | 2008 | ☆ | ☆ |  | ☆ | ☆☆ | ☆ | ☆ | ☆ |
