# Supplementary material for: Evaluating the relationship between right-to-left shunt and white matter hyperintensities in migraine patients: A systematic review and meta-analysis
Source: Front Neurol. 2022 Aug 18;13:972336. doi: 10.3389/fneur.2022.972336 (PMC9433673; doi:10.3389/fneur.2022.972336)
Supplement: Supplementary file 4 [file Table_4.docx]

**Supplementary Table 4 (Cross sectional studies)**

| **Author** | **Year** | **Selection**  **(Max ☆☆☆☆)** | | | | **Comparability**  **(Max ☆☆)** | **Outcome**  **(Max ☆☆☆)** | |
| --- | --- | --- | --- | --- | --- | --- | --- | --- |
|  |  | **Representativeness of the sample** | **Sample size** | **Non-respondents** | **Ascertainment of exposure** | **Comparable cohorts (design or analysis)** | **Assessment of outcome** | **Statistical test** |
| Del Sette, M | 2008 | ☆ | ☆ | ☆ | ☆☆ | ☆☆ | ☆☆ | ☆ |
| Adami, A | 2008 | ☆ | ☆ | ☆ | ☆☆ | ☆☆ | ☆☆ | ☆ |
| Park, H.K. | 2010 | ☆ | ☆ |  | ☆☆ | ☆☆ | ☆☆ | ☆ |
| Iwasaki, A | 2017 | ☆ | ☆ | ☆ | ☆☆ | ☆☆ | ☆☆ | ☆ |
| Xiao-han, J | 2018 | ☆ | ☆ |  | ☆☆ | ☆☆ | ☆☆ | ☆ |
